# Supplementary material for: Heterogeneous wireless networks with mobile devices of multiple interfaces for simultaneous connections using Fuzzy System
Source: PLoS One. 2021 Feb 25;16(2):e0247142. doi: 10.1371/journal.pone.0247142 (PMC7906376; doi:10.1371/journal.pone.0247142)
Supplement: Attachment — Submitted filename: Letter For Reviewers.pdf [file pone.0247142.s001.pdf]

Dear Editor,

First of all, we would like to thank the reviewers for their availability and also to thank all the comments made and that we make every effort to contemplate all. We believe that after the changes made it has improved the quality of the paper.

Reviewer #1: To obtain more bandwidth and avoid competition between user applications on the same wireless technology interface, the manuscript proposes a dual interface device to realize simultaneous use of heterogeneous wireless networks, and a Fuzzy System to select the network for handover in homogeneous network. The simulation results are abundant and interesting, but it still has some problems.

1. The references on network selection are not up to date enough. The papers on network evaluation, selection and switching is scarce and insufficient.

R = New references were included in the related work, in particular, papers [9] e [10].

2. The description of Fuzzy System is not detailed enough and lacks of references. The effect on reducing unnecessary handover has not been explained clearly from simulation results.

R = A new Section was included for explaining Fuzzy. This should provide a better clarification on its functionalities. Also explained in details are possible answers to the systems.

3. In this paper, the authors have mentioned the competition of different applications on the same wireless technology interface. Using two interfaces, the competition may be eliminated when running two applications. But what about the situations running three or more applications such as voice, video and web browsing? A scheme may need to be considered and further explained in this case.

R = A new Section on Major Contributions was included. It explains ping pong handover reduction. Besides, a Figure (and due explanations) was included to show the solution with a middleware between two interfaces. Another Figure was also added to illustrate generic signaling on double handover.

4. Some sections have grammatical mistakes and should be carefully proofread and corrected.

R = A native English speaker has corrected the mistakes and reviewed the entire article again to make the necessary corrections.

Reviewer #2: The authors have produced a thorough investigation into the efficacy of dual interface heterogeneous access technology links for high data rate streaming, in particular for video links. The presented findings demonstrate the advantage of

simultaneously connected heterogeneous access technologies in both link stability and achieving increased overall application level throughput.
It is noted that a comparison is made between a traditional mobile device capable of connecting only to a single access technology and a dual connectivity device with an intelligent handover (fuzzy logic) mechanism.

1. However, it is unclear whether the results presented in figures 5,6,7,8 are representative of average performance or over a single run of the simulation.

R = The information was included in the text and in the Reference Parameter Table.

2. Further to this, although performance over the course of a significant period of time is presented, the statistical distribution of performance across multiple runs hasn't been shown in the case that the behaviour of the fuzzy system in particular is varied under probabilistic wireless channel conditions.

R = In the Major Contribution Section, a text is included to explain that one of the input parameters is RSSI of the interface that detected a new Access Point.

3. Similar concerns are expressed for figures 9,10 and 11 where 'average' performance is presented but it is not clear over how many simulations this average has been taken or whether it is the average performance over a single simulation. Likewise the distribution of these performances has not been indicated if a number of simulations have been performed.

R = This information was included in the text as well as in the Reference Parameters Table.

4. Some minor issues are:
- Table 3 specifies a rate for each of the wireless access technologies in MB (Megabytes) where the interpretation of this may have been intended to be Mbps (Megabits per second).
- The spelling of 'base station' in Figure 1.
- There also appears to be a spelling error in Figure 12 with the spelling of traditional, possibly a mistranslation.

R = All such minor issues were duly corrected.
